# Learning health professionalism at Makerere University: an exploratory study amongst undergraduate students

**DOI:** 10.1186/1472-6920-10-76

**Published:** 2010-11-04

**Authors:** Rhona K Baingana, Noeline Nakasujja, Moses Galukande, Kenneth Omona, David K Mafigiri, Nelson K Sewankambo

**Affiliations:** 1 College of Health Sciences, Makerere University, P. O. Box 7072, Kampala, Uganda; 2 Lira Regional Referral Hospital, P. O. Box 2, Lira, Uganda; 3 Faculty of Social Sciences, Makerere University, P. O. Box 7062, Kampala, Uganda

## Abstract

**Background:**

Anecdotal evidence shows that unprofessional conduct is becoming a common occurrence amongst health workers in Uganda. The development of appropriate professional values, attitudes and behaviors is a continuum that starts when a student joins a health professional training institution and as such health professionals in training need to be exposed to the essence of professionalism. We sought to explore undergraduate health professions students' perceptions and experiences of learning professionalism as a preliminary step in addressing the problem of unprofessional conduct amongst health workers in Uganda.

**Methods:**

Eight focus group discussions were conducted with 49 first to fifth year health professions undergraduate students of the 2008/2009 academic year at Makerere University College of Health Sciences. The focus group discussions were recorded and transcribed, and were analyzed using content analysis with emergent coding.

**Results:**

The difference in the way first and fifth year students of Makerere University College of Health Sciences conceptualized professionalism was suggestive of the decline in attitude that occurs during medical education. The formal curriculum was described as being inadequate while the hidden and informal curricula were found to play a critical role in learning professionalism. Students identified role models as being essential to the development of professionalism and emphasized the need for appropriate role modeling. In our setting, resource constraints present an important, additional challenge to learning universal standards of health professionalism. Furthermore, students described practices that reflect the cultural concept of communalism, which conflicts with the universally accepted standard of individual medical confidentiality. The students questioned the universal applicability of internationally accepted standards of professionalism.

**Conclusions:**

The findings call for a review of the formal professionalism curriculum at Makerere University College of Health Sciences to make it more comprehensive and to meet the needs expressed by the students. Role models need capacity building in professionalism as health professionals and as educators. In our setting, resource constraints present an additional challenge to learning universal standards of health professionalism. There is need for further research and discourse on education in health professionalism in the Sub-Saharan context of resource constraints and cultural challenges.

## Background

Medicine and other health professions are vocations in which professional knowledge, skills and judgment are put in the service of protecting and restoring human well-being. Patients entrust their health in the hands of health workers who are expected to show a high degree of professionalism. Thus professionalism is an essential quality to instill in health professions students, alongside biomedical knowledge and clinical skills in order to produce all-round, competent graduates. Professionalism refers to a collection of attitudes, values, behaviors and relationships that act as the foundation of the health profession's contract with society[1]. Professionalism demands placing the interests of patients above those of the health professional, setting and maintaining standards of competence and integrity, and providing expert advice to society on matters of health[2]. Professionalism was traditionally part of the hidden or informal curriculum. It was "caught rather than taught", and, as an outcome, it was implicit rather than explicit[3]. Of recent, professionalism is considered an explicit learning outcome[4,5], a skill set[6] or a competency[7-9].

Anecdotal evidence shows that public concern about unprofessional conduct among health workers in Uganda is increasing. This is substantiated by studies which indicate that unprofessional conduct among health workers is a major limitation to the provision of quality health services in Uganda. For example, an assessment of community perceptions of quality care in Uganda found that the poor attitude of health workers was characteristic of the services that were perceived as being poor quality[10]. The 2004 World Development Report indicates that as many as 35% of staff were absent from primary health facilities in Uganda[11]. Absenteeism has serious implications as illustrated by an audit of severe maternal morbidity which found that health workers were not available to provide key interventions for 41% of mothers who were found to have received inadequate care[12]. Resale of Government-supplied drugs is a persistent problem that has led to the establishment of a Drug Monitoring Unit[13,14].

The development of appropriate professional values, attitudes and behaviors is a continuum that starts when a student joins a health professional training institution. Hence, we sought to explore undergraduate health professions students' perceptions and experiences of learning professionalism as a preliminary step in addressing the problem of unprofessional conduct amongst health workers in Uganda. Examining students' perceptions of how they learn professionalism can serve as a needs assessment to guide development of a relevant and effective curriculum. Identifying the most appropriate strategies from the learners' perspective will motivate learners to engage in a topic that they may not perceive as essential to the scientific curriculum.

## Methods

### Setting

Makerere University College of Health Sciences (MakCHS) is the oldest and largest health professional training institution in Uganda which offers training in a variety of health care disciplines to about 1500 students through 7 undergraduate and 18 postgraduate programs annually. About 60% of established faculty posts are filled, with 98% at the entry level and 23% at the level of Professor[15]. The main clinical training site is Mulago National Referral Hospital which provides additional clinical teaching staff; however, staff-to-student ratios remain inadequate. The 2009 Human Development Report[16] indicates that government spending on health per capita per year in Uganda is $39 compared to the minimum of $43 needed to provide basic, lifesaving services[17]. Makerere University works on a budget of an average of $1500 per student per year[18] yet the actual cost of training in the health sciences at MakCHS has been estimated at $5,600[19].

The health professions undergraduate programs at MakCHS include a 5-year Bachelor of Medicine and Surgery (MBChB), a 5-year Bachelor of Dental Surgery (BDS), a 4-year Bachelor of Nursing Science (BNS), a 4-year Bachelor of Pharmacy (BPharm) and a 3-year Bachelor of Medical Radiography (BMR). The curricula of these programs were transformed in 2003 from the traditional lecture-based model to a student-centered, problem based and integrated model with community-based education and service, early clinical exposure and electives. Learning is inter-professional in the first two years of study in that the students on the different programs learn together. Students are introduced to professionalism and ethics in the first course of the first year of the undergraduate program. The course is structured around cases/scenarios which lead students within their tutorial groups to discuss key issues in professionalism and ethics such as confidentiality, the doctor-patient relationship, communication skills, etiquette and informed consent. Lectures, role plays and clinical exposure reinforce the learning from tutorial discussions. Besides this, professionalism is expected to be a running theme throughout succeeding years of study of the different programs. The BNS program has a further formal course that discusses nursing ethics and professional issues in nursing. The other undergraduate programs offered by MakCHS are Bachelor of Environmental Health Science (BEHS) and Bachelor of Science in Speech and Language Therapy (BSLT).

### Study population, sample and procedures

The study population consisted of MakCHS clinically-oriented undergraduate health professions students of the 2008/2009 academic year as follows: MBChB (608 students), BDS (68), BNS (68), BMR (35), BPharm (126). The students of BEHS and BSLT were not included because BEHS does not have a clinical component while BSLT is a new program that started only three years ago. Given the homogeneity of the study population per year of study, and that data become "saturated" with little new information emerging after the first few groups [20], we did not anticipate obtaining new information after two focus group discussions for each year of study. Thus, twenty students were selected from each year of study by systematic sampling using student lists stratified by gender and program of study with a view to constituting 2 focus groups of 10 participants per year of study. The 100 selected students were sent a written invitation which also explained the purpose and procedures of the study, including the date, time and venue of the focus group discussions. Forty nine out of the 100 selected students responded to the invitation and participated in 8 focus groups. Students who agreed to participate were offered the opportunity to re-schedule the focus group discussions if the pre-arranged date and/or time were not suitable. Each focus group had 5-8 participants of the same academic year of study. Not all invited students responded to the invitation to participate in the study. Thus, not all programs were represented in each focus group. The distribution by academic program and year of study of students who participated in the study is provided in Table 1.

**Table 1:** Focus group discussion participants by program and year of study

Year	MBChB	BDS	BPharm	BNS	BMR	Total
1	4	-	-	-	1	5
2	5	-	2	1	-	8
3	5	-	-	-	-	5
4	16	4	-	-	NA	20
5	9	2	NA	NA	NA	11

**Total**	**39**	**6**	**2**	**1**	**1**	**49**

NA = Not applicable because the program duration is 3 years (BMR) and 4 years (BPharm, BNS)

A focus group discussion guide was developed by a team of 2 health professionals, 1 biomedical scientist and 1 medical anthropologist. The focus group discussion guide asked participants to discuss:

i. their understanding of professionalism;

ii. whether professionalism applies to them as students;

iii. whether they have learnt (gained knowledge and skills in) professionalism;

iv. how the learning took place.

A team of 4 nonclinical researchers with experience in qualitative research methods and no supervisory relationship to the students conducted the focus group discussions, 2 per focus group; one facilitating the focus group and the second mainly taking notes. The facilitator explained the study to participants at the beginning of each focus group discussion, assured them that comments would not be attributed to them by name and obtained verbal consent for their participation and to record the discussions. The discussions were audio recorded and participants were not identified by name. Participants' responses were later transcribed, and all potential identifiers were removed. The focus group discussions lasted 45-75 minutes. Participants were provided with a soft drink and a snack and did not receive any monetary compensation. The study was carried out in May-June 2009 at the end of the second semester of the 2008/2009 academic year and was approved by Makerere University Faculty of Medicine Research and Ethics Committee.

### Data analysis

Content analysis with emergent coding was used to analyze the data. The study team together analyzed the focus group discussion transcripts line by line for content in order to identify codes by year of study and emerging themes organized by year of study and by the research areas defined in the focus group discussion guide. The elements of teaching and learning professionalism identified by the students were categorized into the formal, informal and hidden curriculum. The formal curriculum is the official approved written curriculum. Following the delineation proposed by Hafferty [21], we defined the hidden curriculum as the physical and workforce organizational infrastructure that influences the learning process, and the informal curriculum as the student's experience of interpersonal processes.

## Results And Discussion

The findings are presented according to the major research topics of the focus group discussion guide as listed above and by the themes that emerged from each research topic. Academic year of study emerged as an important differential, thus, where relevant, student quotations are presented by academic year in order to provide the appropriate perspective. Where necessary, quotations that best represent themes were edited slightly for flow, but the meaning of the text was preserved.

### Conceptualizing professionalism: *"Who I am when I am alone"*

When asked to define professionalism, participants included the following constructs: code of conduct, professional relations, competence, and communication skills (Table 2). In addition, first year students recognised internal motivation as part of professionalism and were idealistic; drawing on morals and self-determination as demonstrated by these responses:

**Table 2:** Concepts of professionalism by year of study

		Year of study				
**Concept**	**Attribute**	**1**	**2**	**3**	**4**	**5**

Internal motivation	Love job	+				
	Re-certification		+			

Good morals	Integrity	+	+		+	
	Kindness	+			+	
	Not ignoring patients			+		

Self-management	Self awareness	+			+	
	Life-long learning	+				
	Knowing limitations, when to refer				+	+

Good communication	Good communication	+	+			+

Code of conduct	Ethics	+	+		+	+
	Hippocratic Oath		+	+		+
	Time keeping	+	+			+
	Respect		+	+	+	+
	Appropriate dress code	+	+	+	+	+
	Privacy		+	+		+
	Confidentiality		+	+	+	+
	Informed consent		+	+		+
	Teamwork	+	+	+		+

Competence	Knowledge	+	+		+	+
	Skills	+	+		+	+

Professional relations	Respect for each other			+	+	+
	Open mind	+			+	+
	Respect for superiors				+	+

The plus sign "+" indicates that the attribute was mentioned by students in the respective year of study. A blank indicates that the attribute was not mentioned.

"In my understanding, who I am when I am alone."

"To be that good person society wants, it starts from within, you can acquire all that knowledge but it's not from within. So I have to work on my character, doing something you do because you love it. It has to start from within."

Idealism amongst first year students has also been reported at health professional training institutions in USA and Canada[22,23]. Exposures such as upbringing and other experiences prior to joining medical school are reported to contribute to idealism by influencing personal values[24] and a student's sense of professional identity[25-27]. Although most students may not be fully grounded in the established, formal professional standards when they enroll at MakCHS, they appear to be motivated by their personal values.

In contrast to the idealism shown by the first years, the perception of the fifth year students was that professionalism is associated with "code of conduct" (Table 2). Their narrative focused on behaviors, not attitudes. The respondents also emphasized the importance of professional relations. The following quotes typify the fifth year concept of professionalism:

"Professionalism is when someone has been trained fully and given knowledge and skills such that they are able to serve using the same knowledge and skills in the best way possible. It's so important because at the end of the day they won't be able to use the skills if professionalism is not practiced so it won't really make sense if the medical personnel are not professionally well equipped."

"In my understanding professionalism is a code of conduct that is required of a practitioner for a particular profession. In health a minimum code of conduct is required to practice where the health practitioner is providing health services or outside the area or institution where he is providing healthcare. There is a minimum standard required for our medical practitioners."

Evidence shows that students experience a longitudinal decline in behaviors and attitudes toward ethical and social issues in medicine as they go through medical school[23,28]. These changes in attitude have not been explored exhaustively, but may relate to loss of idealism and the impact of the hidden and/or informal curriculum. Ours was not a longitudinal study; nonetheless, the divergence in the way first years and the fifth years conceptualized professionalism suggests that the latter have undergone a shift in attitude during their training.

### Relevance of professionalism: *"How can you put on a medical coat and dirty jeans?"*

Participants across all years of study were unanimous that professionalism applies to them as students. They rationalized this in three main ways: firstly that it should be possible to differentiate them from other students by their outward appearance and behavior. This is suggestive of a strong professional identity even among first year students:

"It applies to us because people can know you as a medical student but the way you dress or carry yourself can be a point of shame for example how can you put on a medical coat and dirty jeans?"

Secondly, the quotes below illustrate the students' reasoning that the concepts of professionalism apply to them because they are part of the profession and are being trained in a health care setting:

"So because we are part of the profession it applies to us. Joining medical school means we are part of the system even when we are still students."

"Whatever is happening applies to all of us even the cleaner is part of it so it doesn't disregard anyone."

Lastly, the students explained that they have to be professional from the beginning of their training in order to be professional after qualification; an indication of awareness that the development of professionalism is a continuum and a realization that proper standards of professional behavior have to be sustained for a lifetime:

"They apply to everyone because it starts now and just develops over time, something becomes habitual."

"When you grow in a family where there are norms you just grow up with the norms forever."

"It applies to the students and those already qualified. Most of the things don't change, there's no way you'll do it in the future if you haven't been doing it right now."

The perception held by MakCHS first year students that professionalism is internally motivated by morals and self-determination is shared by first year students at the School of Medicine, University of Minnesota-Duluth, USA (SOMD)[29]. However, unlike MakCHS students who were categorical that professionalism applies to them, SOMD students did not perceive themselves to be obligated by established professional standards. Hafferty[29] surmised that SOMD students either did not know what it means to be a professional as framed within the medical literature or they did not hold to the beliefs and values of the professions. He attributes this to the medical fraternity's communication to the public which is biased towards knowledge and skills which are ranked far above values, attitudes and other issues related to professionalism. While MakCHS students clearly hold to the beliefs and values of the professions, it is beyond the scope of this study to establish the basis of this perception.

### Learning professionalism: *"There has to be a human element who has to be a model"*

Participants identified several elements that are part of teaching and learning professionalism at MakCHS. We categorized them into the formal, hidden and informal curricula (Table 3).

**Table 3:** Components of the Professionalism Curriculum at MakCHS according to 1^st ^to 5^th ^Year Students

Category	Process	Illustrative quotations from transcripts
**Formal**	1^st ^Year course on professionalism	My first tutorial, the tutor was so good, the first thing we dealt with was ethics, he talked about attitude and my attitude wasn't good. I used to be proud, he emphasized the point of respecting and doing good to people; he helped me work on my attitude **(1**^**st **^**Year)** We learnt professionalism theoretically in 1^st ^year, we read up the codes to be followed **(4**^**th **^**Year)**
	
	Lectures	Dr. Y was giving guidance at the very beginning of the course, I got information so lectures helped **(1**^**st **^**Year)**
	
	Reading	My point is that it was gained through reading. We read various books **(1**^**st **^**Year)**

**Hidden**	The tutorial process	In our tutorials, there are students who have better communication skills and we learn from each other **(1**^**st **^**Year)** We conduct our tutorials, we set ground rules which we follow and share information and learn how to interact with fellow students **(2**^**nd **^**Year)**
	
	Experiential learning	Through the wards; the exposure has brought out how we are expected to behave, for example every one knows we need screens before we examine a patient **(4**^**th **^**Year)** First, we learn ethics, then from clinical years, the way you speak to a patient **(5**^**th **^**Year)** It's a people business, no matter what, there's that element of community members, how to deal with them, you must work with people **(5**^**th **^**Year)** I have learnt from class and wards, still on the issue of knowing the limits **(5**^**th **^**Year)**
	
	Community based Education and Service	Interaction with other people when we go for COBES **(2**^**nd **^**Year)** We have never sat in one place, we move in people's districts so we've learnt and are still learning all that is required of us **(4**^th ^Year)

**Informal**	Interaction	Interacting with friends, listening and learning takes place **(3**^**rd **^**Year)** I've learnt confidentiality by keeping my patients' records, I don't just display them, if you don't respect them, if you don't respect patients, you'll never get knowledge **(5**^**th **^**Year)** I have learnt to respect others irrespective of their level of education, that is support staff know things we don't know and communication with patients at every level and social status. **(2**^**nd **^**Year)**
	
	Reflection/self awareness	...but what really impacts itself is what you do, if you can't do that when you are a student, there is no way to do it later on even as a full doctor **(5**^**th **^**Year)** You realize there are things to consider and accepting your limitations. You see how things are done learning from mistakes. It's a process from exposure **(5**^**th **^**Year)**
	
	Role modeling and mentoring	I learnt to respect colleagues from a doctor; he is a senior doctor who gives you time to talk to him; he appreciates every one at the same level **(1**^**st **^**Year)** By examples, so tutors should live as an example and let them go for professionalism courses **(2**^**nd **^**Year)** On the ward when you use what a senior does so you want to do it the same **(3**^**rd **^**Year)** Skills passed on from one to another in medicine can't be learnt in books. There has to be a human element who has to be a model **(4**^**th **^**Year)** The best way to learn is from your seniors, mentoring though it didn't work; 3 students to 1 doctor **(5**^**th **^**Year)**

#### The formal curriculum

While participants appreciated the formal curriculum, they indicated that it was insufficient because it consists of one formal course in the first year of study. The formal curricula of the different programs at MakCHS state that professionalism is a running theme throughout succeeding years of study; however, the challenge has been the operationalization of the curricula. In as much as formal lectures on professionalism provide specific information that may help students interpret what they see on the wards, in the clinics and wherever they may encounter patients, reports indicate that some students see them as lectures on how to behave and therefore find them offensive [24,27]. Others "feel harassed by all of this professionalism talk"[28]. A recent review concludes that didactic coursework alone is insufficient for teaching and learning professionalism issues[30]. Our students made four major suggestions for enriching the formal professionalism curriculum: (1) publish norms of professional behavior for MakCHS students; (2) include issues of professionalism in other courses; (3) institute training activities in the form of workshops and seminars; (4) institute clinical audits. These are in line with the approaches used by some institutions to integrate professionalism into the formal curriculum[24,27]. Other institutions use longitudinal individual assignments which are assessed, and for which students receive feedback[29,31].

#### The hidden curriculum

On the basis of the students' responses, we identified three aspects of the hidden curriculum: the tutorial process, experiential learning, and community based education and service (COBES). It is particularly rewarding that the students recognized the role of the tutorial process and the COBES experience in enhancing skills associated with professionalism such as teamwork and the ability to interact with people from different walks of life (Table 3). Our findings are consistent with other studies which have demonstrated the role of problem-based learning in promoting graduates' interpersonal skills, patient communication and the more general work-related skills that are considered important for success in professional practice[32-34]. There is little documentation about professionalism education in a community-based service-learning context; nevertheless a study in the USA showed that basing a curriculum in community-based organizations is effective in grounding professionalism and professional behavior in the real-world context[35].

We categorized experiential learning as part of the hidden curriculum for professionalism education because even though it is the model used to deliver the formal curriculum for patient care, professionalism has not been formally integrated into experiential learning through the articulation of specific learning objectives and through assessment. Nevertheless, students reported that they learnt about professionalism from being in the wards as patient-care apprentices (Table 3). This finding is in agreement with the results of a study of students at the School of Medicine, University of Washington which suggested that exposure to, and, working directly with patients may promote professionalism, even during the preclinical years[24].

#### The informal curriculum

We identified three elements of the informal curriculum: role modeling and mentoring, reflection, and interaction with peers, senior students, patients and their caregivers (Table 3). One of the consistent themes emerging out of research in health professionalism education is that role models are the primary influence of students' professional development[24,36-39]. Participants from 1^st ^to 5^th ^year reiterated the key part played by role models and the desire for appropriate role models (Table 3). The quote below by a 5^th ^year student vividly illustrates the value of appropriate role modeling:

"Professionalism should be taught or encouraged through role models. I was in the Department of Gynaecology and this woman had tore herself so I just started stitching but this good medical doctor told me that I had to first clean up the patient. The doctor then cleaned the patient in my presence, cleaned her well from head to toe and left me to continue, that was good and from that day I'm challenged."

Our students cited several examples of perceived unprofessional conduct by their teachers and other professionals in the health care setting such as being rude to patients and to students alike, ignoring patients (observed at COBES sites) and clashing with colleagues in front of students and patients. Experiences with both positive and negative behaviors have been found to shape students' perceptions of the profession and its values[38]. Because students' professional development is primarily influenced by role models[24,36-39], repeated negative learning experiences may adversely impact the development of professionalism among health professions students. Furthermore, students' participation in unprofessional conduct has been linked to witnessing unprofessional conduct by peers and by faculty and, what is more, participation in unprofessional behaviors is associated with an enhanced likelihood of viewing the behaviors as acceptable, thus creating a cycle that entrenches unprofessional conduct[27,28]. Role modeling is a fundamental area that has hitherto not been well-exploited at MakCHS where standards pertaining to professionalism are yet to be published.

Reflection is recognized as a core skill in professional competence[3,30,40]. Our students' narratives reveal that they do reflect on their experiences; however, they are not provided with specific opportunities or processes in the curriculum to optimize reflection in the context of professionalism. Reflection transforms experience into understanding and enhances development of professionalism by offsetting the impact of negative role modeling[38]. This is ample justification to integrate reflexive self-examination into the MakCHS curricula.

Interaction with patients was mentioned as one of the ways in which students learn respect for patients and how to communicate with them (Table 3). Early exposure to patients in the teaching hospital setting[41] or the community-based setting[35] is important for development of professionalism because it enables socialization of students into the health profession.

### The learning environment: *"The standards were set for ideal situations"*

The students' responses revealed two important features of the learning environment that operate through the hidden curriculum to influence how they learn professionalism: resource constraints and the cultural context.

#### Resource constraints

In as much as students recognized that professionalism applies to them as individuals, there was the perception that the universally-recognised standards for health professionals may not apply at the institutional level to MakCHS/Mulago National Referral Hospital because prevailing conditions are not ideal:

"The concepts are good but the standards were set for ideal situations, it's not the real situation at hand and you can't explain this to everyone."

The setting at MakCHS/Mulago National Referral Hospital is characterized by high patient numbers compounded by inadequacy of supplies such as screens needed to ensure privacy of patients, and on occasion, gloves to protect both the care giver and the patient. Limitations such as these compel health workers to compromise their professional behavior thus presenting a potential challenge to learning commonly acceptable standards of professionalism and, additionally, causing moral distress among students:

"In the department, there is this particular medical personnel who slapped the mother and yet he can't really be to blame as he had been there the whole night and he was really tired, bad experiences happen, like this one is screaming at you, you see ahead a whole line of patients waiting, it gets frustrating."

"Other areas are difficult in our setting like there are no screens. Sometimes you want to examine, there is no other place to take the patient, and there are no screens."

"As for confidentiality you find that you don't have space, for example in Ward X; beds are together and another patient may hear what we discuss on the ward, you'll never keep confidentiality. It's not the profession but it's the infrastructure."

In her ethnographic study of nursing care at an intensive care unit in Italy, Goopy[42] observed that an over-supply of doctors and a shortage of nurses created "role confusion" for doctors, which led them to encroach on the roles of the nurses. As a result, the nurses did not espouse behaviours and attitudes as expected of their profession such as responsibility and accountability for patient care. The author concluded that a different model of professionalism should be developed for the nurses in this context[42]. Similarly, the observations made by our students challenge the universalistic standards of health professionalism as advanced in the literature and, indeed, by professional councils; the implication is that professionalism standards should be context-specific. This is an issue for debate and further discussion that is beyond the scope of this paper. Whereas Goopy illustrates the impact of resource constraints (shortage of nurses) on professionalism amongst qualified, practicing nurses; the influence of resource constraints on the *development of professionalism before qualification *has not been explored. When teachers cannot ensure a patient's privacy because screens are not available, or when they expose patients to the risk of infection by using the same pair of gloves to examine several patients because gloves are in short supply, they expose students to negative practices. It is not unreasonable to conclude that this will profoundly influence what students learn and eventually practice given that role modelling has a key function in learning professionalism[24,36-39].

#### Cultural context

Like all traditional African cultures, Ugandans have a community-oriented world view as embodied by the philosophy of Ubuntu as reviewed by Ndebele et al.[43]. This means that the concept of individual medical confidentiality that was inherited automatically as part of Western medicine is not in harmony with our culture. Students observed that relatives think they have a right to access patients' information, and moreover, that health care staff actually do give relatives access to patient information even without the patient's consent:

"Relatives think the patient's information must be shared hence breaching confidentiality."

"It's important that patient information is kept confidential and my experience is that it's something that is not done especially with the way medical records are kept; patients' relatives can get in a ward and read patients' files since they are left on beds."

This conflict has been observed in other settings in Africa[43,44] and it is even suggested that the involvement of family members in treatment decisions benefits the individual patient[43]. In addition to communalism there is an authoritarian or paternalistic understanding of relationships in that the health professional is regarded as the expert and regards him/her self as the expert[44]. As a result, patients do not actively demand for information:

"I've noticed that some patients have been discharged without fully knowing what they were suffering from in the first place. The doctors did not take the trouble to explain it to them."

"Medical professionals have read books; we talk many things which will not help the patient. The patient can be in a ward without knowing what she is suffering from and the treatment being used."

Again, the practices described are not congruent with established standards of professionalism. While the paternalistic approach of health professionals in developing countries has been attributed to the low levels of education of most of their patients[44,45], there is some evidence to show that some categories of patient, such as those with progressive incurable disease, and their caregivers, actually want more information[46]. Our students and graduates may not be able to respond to this need for information because they will have been repeatedly exposed to the paternalistic approach practiced by their teachers.

There is an abundance of literature about students' experiences of perceived mistreatment or verbal abuse (see[30] for review) although, to our knowledge, this issue has not been studied at health training institutions in Africa. Our students described similar instances of intimidation, and occasionally, verbal abuse:

"The environment is intimidating, medicine is like the army, it's a silent thing, the system is cruel, most seniors talk as they are moving, we have time but no decision making as he's your senior, he knows terribly much more so its allowed for it to be like a military school. Protocol has to be observed."

"People in offices are supposed to be talking to us but even to get to them, it becomes hard. In 2^nd ^year, one of the doctors exchanged words with me when I told her we were expecting her to teach us......... I thought that wasn't ethical."

It has been suggested that such exposure results in a more cynical attitude towards academic life and to the profession among students[30], the so-called "ethical erosion" or decline in attitudes already discussed[22,23,28,47]. An exposition by students about the experience of learning professionalism in the USA indicates that the chief barrier to medical professionalism education is unprofessional conduct by medical educators, which is protected by an established hierarchy of academic authority[36]. The authors recommend that health professional educators should hold themselves accountable for the unprofessional behavior within the education system. This calls for a standard of educational ethics and professionalization of medical educators as teachers, which is a distinct process from professionalization as health professionals[48].

## Conclusions

In this qualitative study, we explore how health professional students learn professionalism. To the best our knowledge, this is the first time in Africa that students' views about learning health professionalism have been deliberately solicited. Our findings are consistent with findings from North America and Europe that the hidden and informal curricula play a critical role in learning professionalism. In our setting, resource constraints present an additional challenge to learning universal standards of health professionalism. It is noteworthy that the difference in attitude observed between first and fifth year students of MakCHS has also been observed at institutions in North America and Europe, suggesting that resource constraints are not the only factor contributing to the decline in attitude that occurs during medical education. Students described practices which reflect the cultural concept of communalism and which conflict with individual medical confidentiality. These findings draw attention to the need for further research and discourse on health professionalism education in the sub-Saharan African context of resource constraints and cultural challenges.

The findings call for a review of the professionalism curriculum at MakCHS in order to make it more comprehensive and to meet the needs expressed by the students. The curriculum should also provide opportunities to consolidate the personal values that students enroll with as a foundation for instilling the more formal aspects of professionalism. Furthermore, role models need capacity building in professionalism as health professionals and as educators. Professionalism is more than learning and application of technical skills. The inculcation of professional values, attitudes and behaviors requires all members of the health professions to see themselves, and serve, as teachers and exemplars. Professionalism can be learnt through a variety of approaches but most importantly through experience coupled with reflection and discussing exemplary professional behavior. However, if professionalism is to become a more extensive part of any formal curriculum there are many challenges to grapple with including whether and how to assess a student's professionalism.

Given that we did not have baseline or background information in this area relevant to our setting, we carried out an explorative study and as such students were not asked in-depth questions about specific aspects of learning professionalism. Another limitation is that not all students responded to our invitation to participate in the study, thus the medical students were over represented. Thirdly, our study was not designed to longitudinally explore how students learn professionalism. Thus, we are not able to account for the differences between first and fifth year students and can only rely on literature to make inferences.

In seeking students' views through a qualitative approach, we have gained a deeper insight into our curriculum, particularly about the influences outside the formal curriculum. In addition, the open-ended questions enabled students describe aspects of the curriculum that meant the most to them personally. Furthermore, we involved students from the first to the fifth year of study and have thus obtained a panorama of learning professionalism in undergraduates at MakCHS. Lastly, the qualitative and explorative nature of our study was designed to elucidate emerging themes, and it opens the door to future studies.

## Competing interests

NN and MG are health professionals and clinical teachers at MakCHS. RKB tutors first year students at MakCHS. At the time the study was done, KO was out-going President of Makerere University Medical Students' Association. NKS is Principal of Makerere University College of Health Sciences.

## Authors' contributions

RKB, NN, MG, DKM and NKS designed the study. RKB, NN, MG, KO and DKM participated in the development of tools and in the data analysis. RB drafted the manuscript. All authors read and approved the final manuscript. NKS provided critical revision of the manuscript for important intellectual content and gave final approval of the version submitted for publication.

## Pre-publication history

The pre-publication history for this paper can be accessed here:

http://www.biomedcentral.com/1472-6920/10/76/prepub
